# Multimodal assessment of the left ventricular ejection fraction by echocardiography, cardiac computed tomography and cardiac magnetic resonance in patients after SARS-CoV2 infection

**DOI:** 10.3389/fphys.2025.1629065

**Published:** 2025-09-24

**Authors:** Paweł Gać, Andrzej Wysocki, Ewelina Beck, Małgorzata Poręba, Rafał Poręba

**Affiliations:** ^1^ Department of Environmental Health, Occupational Medicine and Epidemiology, Wroclaw Medical University, Wroclaw, Poland; ^2^ Centre of Diagnostic Imaging, 4th Military Hospital, Wroclaw, Poland; ^3^ Department of Biological Principles of Physical Activity, Wroclaw University of Health and Sport Sciences, Wroclaw, Poland

**Keywords:** cardiac computed tomography, cardiac magnetic resonance, echocardiography, left ventricular ejection fraction, SARS-CoV2 infection

## Abstract

**Objective:**

The aim of the study was to compare the assessment of left ventricular ejection fraction (LVEF) performed using echocardiography, cardiac computed tomography (CCT) and cardiac magnetic resonance (CMR) in patients after SARS-CoV2 infection.

**Material and methods:**

The study group consisted of 108 patients (54.17 ± 8.11 years, 52% women and 48% men) with a history of SARS-CoV-2 infection. In all patients, echocardiography, CCT and CMR examinations were performed based on the guidelines of scientific societies. In echocardiography, LVEF (LVEF_ECHO_) was determined from the apical 4-chamber and 2-chamber views, with the biplane Simpson’s method. In CCT, LVEF was assessed based on the contours of the left ventricular endocardium and epicardium in multiplanar reconstructions (MPR) from the multiphase of the entire cardiac cycle, which was part of the protocol of coronary computed tomography angiography performed with retrospective ECG gating with radiation dose modulation (LVEF_CCT1_). Additionally, in CCT, LVEF was assessed based on the left ventricular blood pool in the above reconstructions (LVEF_CCT2_). For the assessment of LVEF in CMR (LVEF_CMR_), a standard volumetric method was used using CINE sequence images in the 2-chamber projection in the long axis and in the short axis of the left ventricle. The coefficient of variation of measurements (CV) was calculated for each pair of LVEF measurements, as well as for all LVEF measurements.

**Results:**

The mean LVEF measurement values in the study group were 59.72% ± 7.39% for LVEF_ECHO_, 63.36% ± 9.32% for LVEF_CCT1_, 64.5% ± 9.79% for LVEF_CCT2_, and 60.84% ± 9.29% for LVEF_CMR_. LVEF_ECHO_ was statistically significantly lower than LVEF_CCT1_ and LVEF_CCT2_. LVEF_CMR_ was also statistically significantly lower than LVEF_CCT1_ and LVEF_CCT2_. CV for all LVEF measurements was 4.61% ± 1.73%. When comparing pairs of LVEF measurements, the lowest CV was observed for LVEF_CCT1_ and LVEF_CCT2_ (2.97% ± 2.64%), while the highest CV was observed for LVEF_ECHO_ and LVEF_CCT2_ (6.04% ± 3.39%). When comparing LVEF to the gold standard of assessment, i.e., LVEF_CMR_, the most consistent measurements were obtained for LVEF_ECHO_ (CV 3.00% ± 2.01%), while the least consistent measurements were obtained for CCT2 (4.65% ± 3.24%). A positive correlation was found between body mass index and CV of LVEF measurements (r = 0.44, p < 0.05), as well as between heart rate (during CCT) and CV of LVEF measurements (r = 0.37, p < 0.05). Furthermore, a negative correlation existed between LVEF measured by ECHO and CV of LVEF measurements in this group of patients (r = - 0.27, p < 0.05).

**Conclusion:**

There are statistically significant differences in left ventricular ejection fraction measurements in patients with a history of SARS-CoV-2 infection using different cardiac imaging modalities. Cardiac computed tomography overestimates LVEF compared to echocardiography and cardiac magnetic resonance imaging. Patients with abnormal body mass, suboptimal heart rate and reduced left ventricular systolic function are subgroups with increased variability of LVEF measurements in different cardiac imaging modalities.

## 1 Introduction

Cardiovascular diseases (CVDs) have remained the predominant cause of global deaths for 30–50 years according to (international) data from the international health organisations. According to the World Health Organisation, in 2019 17.9 million people died from CVDs, which is 32% of all global deaths, whereas in 2021, 20.5 million people died from a cardiovascular condition. Most of these deaths were caused by ischemic heart disease and stroke - major contributors to the global mortality and disability (WHO, 2025). The problem of CVDs is the problem of the entire world population, particularly in low- and middle-income countries and involving prevalent risk factors such as tobacco use, unhealthy diet and obesity, physical inactivity, harmful use of alcohol and air pollution. In the past 3 decades the mortality due to CVDs has been progressively dropping due to targeted health programmes and health promotion. However, the trend is becoming stagnant so to prevent it from reversing further efforts should be made by the health professionals and international institutions to keep it as the priority of public health (World Heart Report, 2023). The consequence of the CVDs might be the development of heart failure (HF) - a life-threatening condition that affects more than 64 million people around the world. For a long time, the general idea of HF was quite elusive so in response a committee of members of the Heart Failure Society of America (HFSA), the Heart Failure Association of the European Society of Cardiology (HFA/ESC), and the Japanese Heart Failure Society (JHFS) suggested the new Universal Definition and Classification of HF in 2021. This definition states that “HF is a clinical syndrome with symptoms and/or signs caused by a structural and/or functional cardiac abnormality and corroborated by elevated natriuretic peptide levels and/or objective evidence of pulmonary or systemic congestion” (Bozkurt et al., 2021). The ESC guidelines define HF quite similarly (McDonagh et al., 2021). According to epidemiological studies, HF is mainly an issue among older adults from well-developed countries. Although the prevalence of HF in the Western countries is falling due to more efficient diagnostics and management, the general prevalence is still rising, ranging between 1% and 3% of the population due to the aging of the global population and increasing number of patients with chronic HF manifestation (Savarese et al., 2023; Roger, 2021). As a matter of fact, epidemiology of the HF is a complex matter regarding the HF classification. HF has been typically characterised by LVEF - a functional parameter describing the ability of the heart to pump blood. This classification distinguishes 3 main phenotypes: HF with reduced (HFrEF, EF ≤ 40%), mildly reduced (HFmrEF, the EF 41%–49%), and preserved EF (HFpEF, ≥50%) (McDonagh et al., 2021; Heart Organization, 2025). This division appears to be efficient from the clinical point of view by facilitating the right therapy choice according to the HF type. On an important note, one can develop heart failure while maintaining normal EF also known as a heart failure with preserved ejection fraction (HFpEF). As mentioned, the left ventricle ejection fraction (LVEF) is a parameter corresponding with the function of the left ventricle, thus cardiac function. It is a volumetric parameter expressed as a percentage of blood volume ejected from the LV in each cardiac cycle. In other words, it might be defined as a ratio of stroke volume to end-diastolic volume (Kerkhof et al., 2018). Traditionally the LVEF has been used to classify heart failure into phenotypes - each of them targeted by specific treatment - and establish prognosis (Marwick, 2018). However, over the recent years its role as a reliable marker of the LV function has been questioned and widely discussed. Some authors proposed alternative parameters to assess LV function such as global longitudinal strain (GLS). Although the paradigm of LVEF has been questioned, it has kept its place in certain areas of cardiology as a diagnostic and prognostic factor. One should be mindful of its limitations, alternating factors as well as assessment methods along with technical details, which will allow one to understand why LVEF calculations may differ among modalities and what affects LVEF repeatability (Diaz-Navarro and Kerkhof, 2024).

In practice, LVEF can be assessed by different modalities including echocardiography, cardiac computed tomography and cardiac magnetic resonance, using either subjective visual or objective quantitative methods (Kosaraju et al., 2025). Each modality has its strengths and limitations, which directly affects the results of the LVEF measurements and repeatability (Foley et al., 2012). Currently, cardiac magnetic resonance is the “golden standard” of assessment of LVEF due to excellent contrast resolution and high repeatability. At the same time, CMR images should be evaluated by an experienced radiologist specialised in cardiac imaging for the most accurate result (Tański et al., 2021; Salerno et al., 2017).

Undoubtedly, cardiac imaging fulfilled its task during COVID-19 pandemics, leading to a better understanding of the mechanism of cardiovascular complications and long-term effects of SARS-CoV2 infection (Holby et al., 2023). Cardiac imaging techniques provided crucial information on pathomechanism of cardiac injury and allowed post-infection follow-up. Particularly echocardiography and CMR turned out to be useful modalities. Investigated cardiac manifestations of COVID-19 include myocarditis, myocardial ischemia or infarction, heart failure, arrhythmias and arterial or venous thromboembolism (Crosier et al., 2023). DELIVER trial (Dapagliflozin Evaluation to Improve the Lives of Patients with Preserved Ejection Fraction Heart Failure trial), a very comprehensive trial among patients with chronic heart failure with mildly reduced or preserved ejection fraction (HFmrEF/HFpEF) who were randomized to dapagliflozin or placebo across 350 sites in 20 countries showed that mean LVEF was similar between those who did (54.1 ± 8.4%) and did not (54.2 ± 8.8%) develop COVID-19 (Bhatt et al., 2023). However, it has been proved that COVID-19 may lead to secondary ventricular dysfunction, although the ventricular dysfunction was more obvious in patients with cardiovascular comorbidities, including heart failure, and already elevated troponin levels (Artico et al., 2023; Chung et al., 2021). In a report published in JAMA Cardiology researchers from Germany examined data from 100 patients recovered from COVID-19 after 2–3 months after their COVID-19 diagnosis. It turned out that the recovered patients had greater left ventricular volume and lower ejection fraction in comparison to a control group (Abbasi, 2021).

Numerous complications, including cardiovascular complications, have been described during COVID-19. Therefore, a reliable assessment of cardiac function, primarily through a reliable assessment of left ventricular ejection fraction, appears necessary in certain clinical situations in patients with a history of COVID-19 (Artico et al., 2023; Chung et al., 2021). These include: patients with symptoms suggestive of cardiac injury (shortness of breath, easy fatigability, oedema, chest pain, palpitations), patients with a history of severe COVID-19, especially those with myocarditis or cardiovascular involvement, patients with elevated markers of cardiac injury (e.g., troponin, NT-proBNP), individuals with previously diagnosed heart disease (e.g., heart failure, cardiomyopathy), and patients requiring cardiac function assessment before returning to intense physical activity or sports.

The aim of the study was to compare the assessment of left ventricular ejection fraction (LVEF) performed using echocardiography, cardiac computed tomography (CCT) and cardiac magnetic resonance (CMR) in patients after SARS-CoV2 infection.

## 2 Materials and methods

The study was conducted as part of the Wroclaw Medical University project no. SUBZ.E264.23.039 entitled “The importance of selected laboratory, imaging, and electrophysiological diagnostic methods in the assessment of cardiovascular health.” All procedures performed in the study involving human participants were in accordance with the ethical standards of the Ethics Committee of Wroclaw Medical University and with the 1964 Helsinki Declaration and its later amendments or comparable ethical standards. The study protocol was approved by the Ethics Committee of Wroclaw Medical University (consent no. ID KB-210/2023, date of approval: 9 March 2023).

The study group consisted of 108 patients. All patients had symptoms of SARS-CoV-2 infection. 62.0% of patients were hospitalized due to COVID-19, including 12.0% in the intensive care unit. The remaining patients were treated on an outpatient basis. The mean age of the study group was 54.17 ± 8.11 years. Both genders were enrolled in the research (52% women and 48% men). The mean body mass index (BMI) was 27.16 ± 2.90, which was calculated using the formula BMI = body weight [kg]/height [m]^2^. Out of the entire study group, only 22.2% of the patients had normal BMI. 62.0% of patients were overweight, and 15.7% were obese. Additionally, the protocol of the study involved the coexistence of cardiovascular risk factors, such as arterial hypertension (40.7%), type 2 diabetes (11.1%), dyslipidaemia (46.7%) and smoking (32.4%). The general characteristics of the study group are presented in Table 1.

**TABLE 1:** Clinical characteristics of the study group.

Parameter	Whole study group (n = 108)
Age [years]^a^	54.17 ± 8.11
Gender^b^	
Men	48.1
Women	51.8
BMI [kg/m^2^]^a^	27.16 ± 2.90
Overweight/obesity^b^	
Normal body mass	22.2
Overweight	62.0
Obesity	15.7
coexistence of cardiovascular risk factors^b^	
Arterial hypertension	40.7
Type 2 diabetes	11.1
Dyslipidemia	46.7
Smoking	32.4
Symptomatic SARS-CoV-2 infection^b^	100.0
Hospitalization due to infection^b^	62.0
hospitalization in the ICU due to infection^b^	12.0

^a^ quantitative variable expressed as mean ± standard deviation.

^b^ categorical variable expressed as percentage.

BMI, body mass index; ICU, intensive care unit.

In the study, transthoracic echocardiography was performed using the ALOKA ProSound SSD-5500 SV, equipped with a 3.5/2.7 MHz transducer (Aloka Inc., Tokyo, Japan). The results were evaluated using the mentioned criteria of the American Society of Echocardiography. LVEF (LVEF_ECHO_) was determined from the apical 4-chamber and 2-chamber views, with the biplane Simpson’s method. The American Society of Echocardiography recommends the modified Simpson’s rule (also known as the biplane method of disks) as a 2D method to assess LVEF. Two sets of LV measurements were obtained in the apical 4-chamber and 2-chamber projections by tracing the endocardial border in the end-systole and end-diastole, then the LV cavity was subdivided into dimensional discs. Ventricular end-diastolic diameter (LVEDd), left ventricular end-systolic diameter (LVESd), interventricular septum diastolic diameter (IVSDd) and posterior wall diastolic diameter (PWDd) were obtained using M-mode and Penn convention, with an accuracy of 1 mm. Then, LVEF was calculated based on EDV and ESV following the formula: EF = EDV-ESV/EDV × 100%.

The cardiac computed tomography (CCT) was conducted following the standard coronary CT angiography (CCTA) protocol with the use of a dual-source 384-slice CT scanner SOMATOM Force (Siemens Healthcare, Erlangen, Germany). The acquired images were evaluated by a certified radiologist with an EACVI Cardiac Computed Tomography Exam certification and more than 10 years of clinical experience. During the examination an intravenous contrast medium was administered at a volume determined by the patient’s body weight to obtain high-resolution images, differentiating between the LV cavity and the endocardium. Images were obtained with a single breath hold. Standard evaluation of the CCT examination using the CAD-RADS (Coronary Artery Disease-Reporting and Data System) classification system was performed. According to CAD-RADS, 0 documented absence of coronary artery disease (CAD), 1 - minimal nonobstructive CAD (maximal stenosis: 1%–24%), 2 -mild nonobstructive CAD (maximal stenosis: 25%–49%), 3 - moderate CAD (maximal stenosis: 50%–69%), 4 - severe CAD (maximal stenosis: 70%–99%), and 5- total coronary artery occlusion. In CCT, LVEF was assessed based on the contours of the left ventricular endocardium and epicardium in multiplanar reconstructions (MPR) from the multiphase of the entire cardiac cycle (in the range from 0% to 100% of the cycle duration, in steps of 10%), which was part of the protocol of coronary computed tomography angiography performed with retrospective ECG gating with radiation dose modulation (LVEF_CCT1_). Additionally, in CCT, LVEF was assessed based on the left ventricular blood pool in the above reconstructions (LVEF_CCT2_). The standard mode includes the papillary muscles in the calculation, whereas they are excluded in the blood volume mode. While the standard mode analyzes 2D contrast-enhanced CT slices to calculate end-diastolic and end-systolic volumes, then computes LVEF, the blood volume mode uses 3D reconstructions from CT images for identifying and tracking the movement of the heart’s walls, for more accurate measurements of end-diastolic and end-systolic volumes. Syngo. CT Cardiac Function instantly processed the data to perform automated segmentation of the ventricles and then calculated global parameters like ejection fraction, myocardial mass, stroke volume, end-systolic, and end-diastolic volumes.

The CMR (Cardiac Magnetic Resonance) tests were performed using a 1.5 T Magnetom Aera (Siemens Healthcare) according to a standardized protocol, including ECG-gated imaging with breath holds. The imaging sequences used were CINE-type SSFP, STIR, and LGE sequences, with gadobutrol (Gadovist) injection for LGE imaging. Cine imaging was performed in LV 2-, 3-, and 4-chamber apical views as well as in short-axis views encompassing the entire LV and right ventricular (RV) myocardium using balanced steady-state free precession (SSFP) gradient echo (slice/gap thickness: 10/0 mm, matrix: 256 × 192, in-plane resolution: 1.4 × 1.4 mm^2^; repetition time (TR)/echo time (TE): 3.0/1.3 ms, flip angle: 59°, parallel imaging technique (generalized autocalibrating partially parallel acquisition GRAPPA, number of reconstructed phases per cardiac cycle: 30). The 4-chamber projection measured the left and right atrial surface areas (LAA, RAA), while the short axis projection assessed LVEDD (left ventricular end-diastolic diameter), LVESD (left ventricular end-systolic diameter), IVS-EDWT (interventricular septal end‐diastolic wall thickness) and PW-EDWT (posterior wall end‐diastolic thickness). For the assessment of LVEF in CMR (LVEF_CMR_), a standard volumetric method was used using CINE sequence images in the 2-chamber projection in the long axis and in the short axis of the left ventricle. Currently CMR is the “golden standard” of assessing left ventricular function. EDV and ESV were calculated from short axis images by summing the left ventricular cavity surface area across layers. Stroke volume (SV) was the difference between EDV and ESV, and ejection fraction (EF) was calculated by: EF = SV/EDV*100%. These functional parameters were indexed to body surface area (BSA), and LV mass index (LVMI) was also determined. STIR and LGE sequences were used to assess myocardial morphological changes, including detecting oedema and LGE foci in the left and right ventricles. The presence and size of oedema were evaluated using the T2 ratio (myocardial intensity vs. skeletal muscle intensity). A T2 ratio greater than 1.9 indicated generalized oedema. LGE was classified based on its location (transmural, sub-epicardial, intramural, subendocardial). The presence of intramural or subendocardial LGE indicated ischemic injury, while sub-epicardial or intramural LGE pointed to non-ischemic injury. The presence of pericardial fluid was assessed.

In this study, the authors essay to assess the repeatability of the LVEF measurements by different modalities. The analysis of repeatability was conducted involving quantitative variables, calculated on the basis of the measurements of the LVEF for each modality, then correlating them with one another and ultimately comparing LVEF in each modality with the gold standard of assessment, i.e., LVEF_CMR_. The results were analyzed independently by 2 radiologists experienced in the evaluation of the cardiovascular system. The analysis regarded variables including measurement mean (X), standard deviation (SD) and coefficient of measurement variation (CV). To calculate the latter the following mathematical formula was used: CV = SD of the measurement/X of the measurement × 100. The CV was expressed as a percentage. The coefficient of variation of measurements (CV) was calculated for each pair of LVEF measurements, as well as for all LVEF measurements. The consistency of measurements was based on the gold standard of LVEF assessment - LVEF_CMR_.

Statistical analysis was carried out using Dell Statistica v. 13 (Dell Inc., Austin, TX, United States). For numerical data, we reported the mean and standard deviation (SD). The Shapiro–Wilk test was used to check if the data followed a normal distribution. The statistical significance of differences in EF measurements was assessed using ANOVA for repeated measures. Categorical data were shown as numbers and percentages. To examine relationships between variables, we used correlation analysis for two variables and multiple regression for more complex comparisons. Values of p < 0.05 were considered statistically significant.

## 3 Results

Table 2 presents the echocardiographic results in the entire group. The mean left ventricular ejection fraction (LVEF) in ECHO was 59.72% ± 7.39% (minimum: 40%, maximum: 70%). Left and right ventricular end-diastolic diameters were 53.61 ± 6.17 mm and 23.39 ± 2.90 mm, respectively, and left atrial diameter was 38.59 ± 3.72 mm. The mean left ventricular myocardial thickness in the study group was normal, measuring 9.96 ± 0.96 mm in diastole for the interventricular septum and 10.05 ± 1.10 mm for the posterior wall of the left ventricle.

**TABLE 2:** Basic parameters measured by echocardiography in the study group.

Parameter	Whole study group (n = 108)
LVEDD [mm]^a^	53.61 ± 6.17
LVESD [mm]^a^	33.51 ± 6.23
IVSEDD [mm]^a^	9.96 ± 0.96
PWEDD [mm]^a^	10.05 ± 1.10
RVEDD [mm]^a^	23.39 ± 2.90
LA [mm]^a^	38.59 ± 3.72
Ao [mm]^a^	30.13 ± 3.95
LVEF [%]^a^	59.72 ± 7.39

^a^ quantitative variable expressed as mean ± standard deviation.

Ao, ascending aorta diameter, IVSEDD, interventricular septum end-diastolic diameter, LA, left atrium diameter; LVEDD, left ventricular end-diastolic diameter; LVEF, left ventricular ejection fraction; LVESD, left ventricular end-systolic diameter; PWEDD, posterior wall end-diastolic diameter; RVEDD, right ventricular end-diastolic diameter.

Table 3 outlines basic coronary computed tomography angiography parameters in the study group, which are coronary artery calcium score (CACS) and Coronary Artery Disease-Reporting and Data System (CAD-RADS). The average CACS obtained in the whole group of patients was 240.60 ± 249.76, which corresponds with moderate risk of significant coronary artery disease. In the study group, 30.6% of patients were scored as CAD-RADS 0, 13.0% as 1, 25.0% as 2, 22.22% as 3, 6.5% as 4 and finally 2.7% of patients were assessed as 5. Depending on the measurement method, the left ventricular ejection fraction assessed by computed tomography was 63.36% ± 9.32% (minimum: 42%, maximum: 78%, when measured based on the contours of the LV endocardium) and 64.50% ± 9.79% (minimum: 35%, maximum: 81%, when measured based on the size of the left ventricular blood pool).

**TABLE 3:** Basic coronary computed tomography angiography parameters in the study group.

Parameter	Whole study group (n = 108)
CACS^a^	240.60 ± 249.76
CAD-RADS^b^	
0	30.6
1	13.0
2	25.0
3	22.2
4	6.5
5	2.7
LVEF [%]^a^	
Assessed based on the contours of LV endocardium (CCT1)	63.36 ± 9.32
Assessed based on LV blood pool (CCT2)	64.50 ± 9.79

^a^ quantitative variable expressed as mean ± standard deviation.

^b^ categorical variable expressed as percentage.

CACS, coronary artery calcium score; CAD, coronary artery diseases; CCT, cardiac computed tomography; LV, left ventricle; LVEF, left ventricular ejection fraction; RADS, reporting and data system.

CMR examination in morphological sequences revealed the presence of left ventricular myocardial injury in 4.6% of patients, including 0.9% with ischemic injury and 3.7% with non-ischemic injury. No patient showed evidence of left ventricular myocardial oedema. The mean LVEF representing left ventricular function was 60.84% ± 9.29% (minimum: 39%, maximum: 77%). In summary, cardiac magnetic resonance parameters in the study group have been summarized in Table 4.

**TABLE 4:** Basic cardiac magnetic resonance parameters in the study group.

Parameter	Whole study group (n = 108)
LAA [cm^2^]^a^	27.51 ± 3.50
RAA [cm^2^]^a^	21.22 ± 2.56
LVEDD [mm]^a^	56.75 ± 7.83
LVESD [mm]^a^	35.04 ± 8.27
IVS-EDWT [mm]^a^	8.93 ± 6.54
PW-EDWT [mm]^a^	8.40 ± 1.14
LVMI [g/m^2^]^a^	73.19 ± 11.72
LVEDVI [ml/m^2^]^a^	85.49 ± 19.71
LVESVI [ml/m^2^]^a^	37.19 ± 11.49
LVSVI [ml/m^2^]^a^	47.19 ± 8.84
LVEF [%]^a^	60.84 ± 9.29
LV myocardium edema^b^	0.0
LV myocardium LGE^b^	4.6
LV myocardium ischemic injury^b^	0.9
LV myocardium nonischemic injury^b^	3.7
pericardial effusion^b^	13.0

^a^ quantitative variable expressed as mean ± standard deviation.

^b^ categorical variable expressed as percentage.

IVS-EDWT, interventricular septal end‐diastolic wall thickness, LAA, left atrial area; LGE, late gadolinium enhancement; LV, left ventricular; LVEDD, left ventricular end-diastolic diameter; LVEDVI, left ventricular end-diastolic volume index; LVEF, left ventricular ejection fraction; LVESD, left ventricular end-systolic diameter; LVESVI, left ventricular end-systolic volume index; LVMI, left ventricular mass index; LVSVI, left ventricular stroke volume index; PW-EDWT, posterior wall end‐diastolic thickness; RAA, right atrial area.

In Table 5 the authors present the results of the multimodal assessment of the left ventricular ejection fraction by three different modalities - echocardiography, cardiac computed tomography and cardiac magnetic resonance. LVEF_ECHO_ was 59.72% ± 7.39%. In CCT, LVEF_CCT1_ and LVEF_CCT2_ were 63.36% ± 9.32% and 64.50% ± 9.79%, respectively. LVEF _CMR_ was 60.84% ± 9.29%. LVEF_ECHO_ was statistically significantly lower than LVEF_CCT1_ and LVEF_CCT2_. LVEF_CMR_ was also statistically significantly lower than LVEF_CCT1_ and LVEF_CCT2_.

**TABLE 5:** Multimodal assessment of the left ventricular ejection fraction by echocardiography, cardiac computed tomography and cardiac magnetic resonance.

Parameter	Whole study group (n = 108)	p < 0.05
LVEF _ECHO_ [%]^a^	59.72 ± 7.39	LVEF _ECHO_ vs. LVEF _CCT1_ LVEF _ECHO_ vs. LVEF _CCT2_ LVEF _CCT1_ vs. LVEF _CMR_ LVEF _CCT2_ vs. LVEF _CMR_
LVEF _CCT1_ [%]^a^	63.36 ± 9.32	
LVEF _CCT2_ [%]^a^	64.50 ± 9.79	
LVEF _CMR_ [%]^a^	60.84 ± 9.29	
CV for LVEF [%]^a^	4.61 ± 1.73	CV for LVEF _ECHO_ vs. LVEF _CCT2_ vs. CV for LVEF _CCT1_ vs. LVEF _CCT2_ CV for LVEF _ECHO_ vs. LVEF _CCT2_ vs. CV for LVEF _ECHO_ vs. LVEF _CMR_ CV for LVEF _ECHO_ vs. LVEF _CCT2_ vs. CV for LVEF _CCT1_ vs. LVEF _CMR_ CV for LVEF _CCT1_ vs. LVEF _CCT2_ vs. CV for LVEF _CCT2_ vs. LVEF _CMR_
CV for LVEF _ECHO_ vs. LVEF _CCT1_ [%]^a^	4.36 ± 2.57	
CV for LVEF _ECHO_ vs. LVEF _CCT2_ [%]^a^	6.04 ± 3.39	
CV for LVEF _ECHO_ vs. LVEF _CMR_ [%]^a^	3.00 ± 2.01	
CV for LVEF _CCT1_ vs. LVEF _CCT2_ [%]^a^	2.97 ± 2.64	
CV for LVEF _CCT1_ vs. LVEF _CMR_ [%]^a^	3.06 ± 1.49	
CV for LVEF _CCT2_ vs. LVEF _CMR_ [%]^a^	4.65 ± 3.25	

^a^ quantitative variable expressed as mean ± standard deviation.

CV, coefficient of variation of measurements; CCT, cardiac computed tomography; CMR, cardiac magnetic resonance; ECHO, echocardiography; LVEF, left ventricular ejection fraction.

Then, LVEF measurements by all three modalities were paired together and compared with one another. CV was measured for all LVEF measurements and each pair of them. CV for all LVEF measurements was 4.61% ± 1.73%. When comparing pairs of LVEF measurements, the lowest CV was observed for LVEF_CCT1_ and LVEF_CCT2_ (2.97% ± 2.64%), while the highest CV was observed for LVEF_ECHO_ and LVEF_CCT2_ (6.04% ± 3.39%). When comparing LVEF to the gold standard of assessment, i.e., LVEF_CMR_, the most consistent measurements were obtained for LVEF_ECHO_ (CV 3.00% ± 2.01%), while the least consistent measurements were obtained for CCT2 (4.65% ± 3.24%).

In the study correlation analysis was conducted to determine relationships between values of the left ventricular ejection fraction assessed by different cardiac imaging modalities. Correlation coefficient (r) was calculated for each pair of modalities. For all paired modalities, r ranged from 0.93 to 0.99, which implies a strong positive linear relationship between the measurements. Thus, the research has shown strong positive correlations, which corresponds with r close to 1. The highest r was determined for CCT1 and CMR, whereas the lowest for ECHO and CCT2. Despite minor differences the results of LVEF measurements using different cardiac imaging modalities appear similar. Table 6 showcases the outcomes from the correlation study.

**TABLE 6:** Results of correlation analysis of left ventricular ejection fraction assessed by different cardiac imaging modalities.

Parameter	LVEF _ECHO_ [%]	LVEF _CCT1_ [%]	LVEF _CCT2_ [%]	LVEF _CMR_ [%]
LVEF _ECHO_ [%]	1.00	0.96	0.93	0.96
LVEF _CCT1_ [%]	0.96	1.00	0.95	0.99
LVEF _CCT2_ [%]	0.93	0.95	1.00	0.95
LVEF _CMR_ [%]	0.96	0.99	0.95	1.00

CCT, cardiac computed tomography; CMR, cardiac magnetic resonance; ECHO, echocardiography; LVEF, left ventricular ejection fraction.

Finally, a regression analysis was performed for LVEF values measured by different modalities to model the relationship between two variables. Parameters considered in the model included estimated measurement of LVEF in one modality and known measurement of LVEF, both expressed as percentage. In this case, the independent variable (e.g., LVEF measured by ECHO) is used to predict the dependent variable (e.g., LVEF measured by CCT1, CCT2, or CMR). The obtained mathematical equations indicate linear relationships between modalities. The regression coefficients (e.g., 1.22, 0.76) represent the slope of the relationship, and the constant terms (e.g., −9.28, +11.25) represent the offset. Detailed results of the regression analysis in the study group are presented in Table 7. From the clinical point of view, the calculated formulas create a practical tool in settings where one imaging modality is preferred due to factors like availability, patient condition, or cost. In scientific research they might be useful when comparing results across imaging techniques.

**TABLE 7:** Mathematical formulas enabling the estimation of the left ventricular ejection fraction in each modality based on the values measured using another modality in the study group, obtained in the regression analysis.

Parameters considered in the model	Mathematical equation
estimated measurement: LVEF _CCT1_ [%] known measurement: LVEF _ECHO_ [%]	LVEF _CCT1_ [%] = 1.22 LVEF _ECHO_ [%] – 9.28
estimated measurement: LVEF _CCT2_ [%] known measurement: LVEF _ECHO_ [%]	LVEF _CCT2_ [%] = 1.23 LVEF _ECHO_ [%] – 8.80
estimated measurement: LVEF _CMR_ [%] known measurement: LVEF _ECHO_ [%]	LVEF _CMR_ [%] = 1.21 LVEF _ECHO_ [%] – 11.43
estimated measurement: LVEF _ECHO_ [%] known measurement: LVEF _CCT1_ [%]	LVEF _ECHO_ [%] = 0.76 LVEF _CCT1_ [%] + 11.25
estimated measurement: LVEF _CCT2_ [%] known measurement: LVEF _CCT1_ [%]	LVEF _CCT2_ [%] = 1.01 LVEF _CCT1_ [%] + 1.09
estimated measurement: LVEF _CMR_ [%] known measurement: LVEF _CCT1_ [%]	LVEF _CMR_ [%] = 0.98 LVEF _CCT1_ [%] – 1.59
estimated measurement: LVEF _ECHO_ [%] known measurement: LVEF _CCT2_ [%]	LVEF _ECHO_ [%] = 0.70 LVEF _CCT2_ [%] – 14.63
estimated measurement: LVEF _CCT1_ [%] known measurement: LVEF _CCT2_ [%]	LVEF _CCT1_ [%] = 0.91 LVEF _CCT2_ [%] + 4.91
estimated measurement: LVEF _CMR_ [%] known measurement: LVEF _CCT2_ [%]	LVEF _CMR_ [%] = 0.90 LVEF _CCT2_ [%] +2.61
estimated measurement: LVEF _ECHO_ [%] known measurement: LVEF _CMR_ [%]	LVEF _ECHO_ [%] = 0.76 LVEF _CMR_ [%] + 13.16
estimated measurement: LVEF _CCT1_ [%] known measurement: LVEF _CMR_ [%]	LVEF _CCT1_ [%] = 0.99 LVEF _CMR_ [%] + 3.07
estimated measurement: LVEF _CCT2_ [%] known measurement: LVEF _CMR_ [%]	LVEF _CCT2_ [%] = 1.01 LVEF _CMR_ [%] + 3.51

CCT, cardiac computed tomography; CMR, cardiac magnetic resonance; ECHO, echocardiography; LVEF, left ventricular ejection fraction.

**FIGURE 1:**
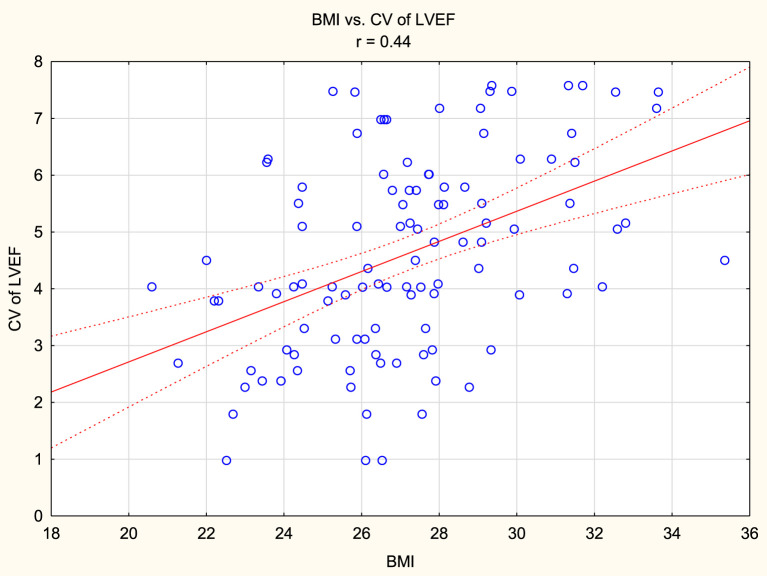
Positive correlation between body mass index (BMI) and coefficient of variation (CV) of left ventricular ejection fraction (LVEF) measurements (r = 0.44, p < 0.05).

**FIGURE 2:**
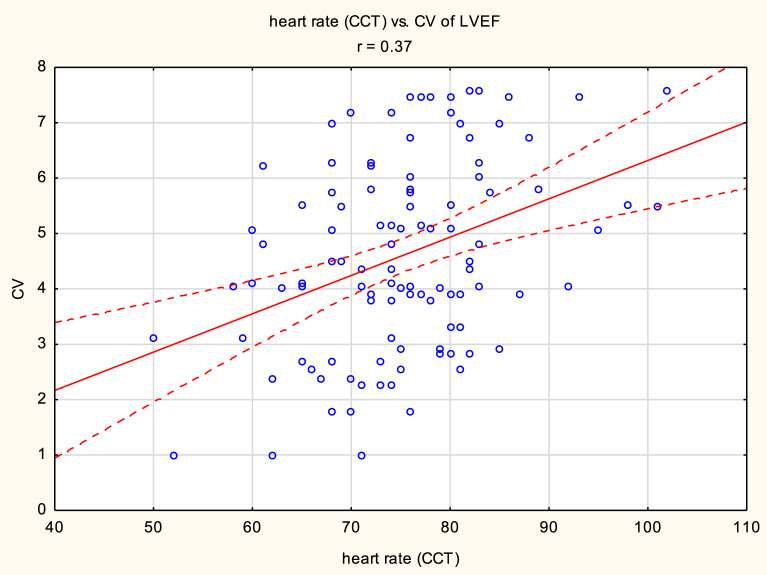
Positive correlation between heart rate during cardiac computed tomography (CCT) and coefficient of variation (CV) of left ventricular ejection fraction (LVEF) measurements (r = 0.37, p < 0.05).

**FIGURE 3:**
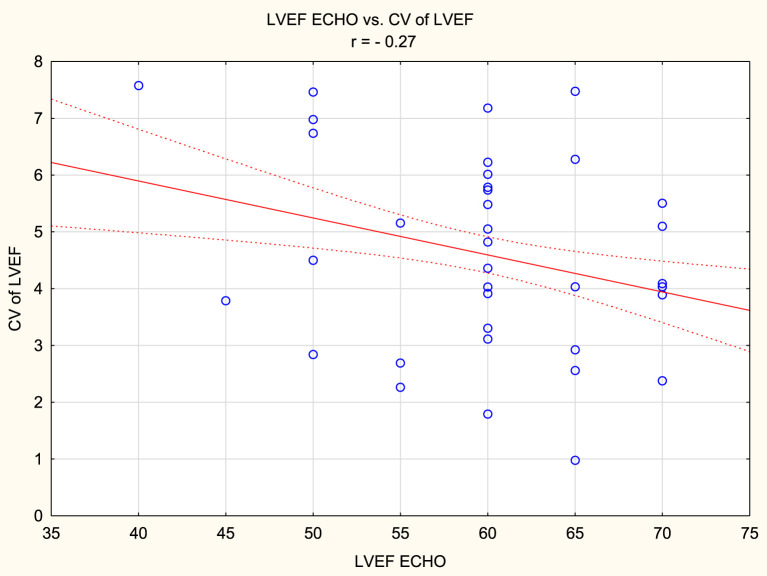
Negative correlation between left ventricular ejection fraction (LVEF) measured by ECHO and coefficient of variation (CV) of LVEF measurements (r = - 0.27, p < 0.05).

A positive correlation was found between body mass index and CV of LVEF measurements (r = 0.44, p < 0.05), as well as between heart rate (during CCT) and CV of LVEF measurements (r = 0.37, p < 0.05). Furthermore, a negative correlation existed between LVEF measured by ECHO and CV of LVEF measurements in this group of patients (r = - 0.27, p < 0.05). Figures 1–3 presents the above statistically significant correlations.

## 4 Discussion

As mentioned, EF might not be the perfect parameter of the ventricular function but still it has a significant application in contemporary cardiology. At the same time, cardiac imaging modalities have advanced, and each modality has inherent sources of error, and the choice of the most suitable method depends largely on the clinical context (Foley et al., 2012).

In our study, we compared left ventricular ejection fraction (LVEF) assessed by three different modalities—Echocardiography (ECHO), Cardiac Computed Tomography (CCT), and Cardiac Magnetic Resonance (CMR)—in patients who had previously been infected with SARS-CoV-2. The results showed that there are statistically significant differences in left ventricular ejection fraction measurements in patients with a history of SARS-CoV-2 infection using different cardiac imaging modalities. Cardiac computed tomography overestimates LVEF compared to echocardiography and cardiac magnetic resonance imaging. The observed discrepancies primarily resulted from technical variations between the modalities, differences in the assumptions made during the calculations of LVEF, and inter-individual variability in patients, such as differences in body mass, heart rate, and the presence of reduced LVEF.

Numerous studies have compared the accuracy and repeatability of LVEF measurements across ECHO, CCT, and CMR, aiming to identify the correlations between them as well as factors determining the repeatability of LVEF measurements. In most cases, the LVEF measurements were discussed in reference to the CMR as the golden standard of measurement. In 2013, Wood et al. performed a comprehensive review of the research (2 multicenter, 16 single center) on measurements of left ventricular (LV) volume and ejection fraction (LVEF) from two-dimensional (2D ECHO) and three-dimensional (3D ECHO) echocardiography, nuclear imaging, cardiac computed tomography, and cardiac magnetic resonance imaging (CMR) (Wood et al., 2014). In their study, they reported discrepancies mainly in measurements of left ventricular (LV) volume and ejection fraction (LVEF) and only minor differences in LVEF in studies comparing CMR and 2D contrast echocardiography or noncontrast 3D echocardiography. They also found that both 2D and 3D ECHO tended to underestimate LV volumes as well as indicated distinct variability compared to those delivered by CMR. Most studies that assessed LVEF using CT reported remarkable variability - the measurements of LVEF from these studies were consistently reliable and reproducible across different subjects and situations. CT was considered to have high accuracy due to border delineation and volume calculation techniques like those used in Cardiac Magnetic Resonance (CMR). By using higher spatial resolution, CT can offer similar performance to CMR when it comes to determining left ventricular volumes and ejection fraction. Another review, by Kinno et al. from 2017 discussed and compared Echo, CMR, and MDCT for the assessment of systolic and regional left ventricular function in reference to other authors (Kinno et al., 2017). Again, CMR was referred to as the golden standard method - it presented low interobserver variability and low intraobserver variability for the assessment of EF (ranging from 5.1% to 6.3% and 3.7%–5.7% respectively). Meta-analysis of 23 studies by Dorosz et al. presented that differences in biases between 2D and 3D Echo-derived values were significant for LV volumes, but not for EF in comparison to CMR (Dorosz et al., 2012). Then, Hoffman et al. showed that LVEF derived from contrast-enhanced 2D or 3D Echo were similar with CMR and more accurate than non-contrast-enhanced 2D and 3D Echo (Hoffmann et al., 2005). On the other hand, Sarwar et al. compared the LV global assessment by contrast-enhanced 64-slice MDCT with CMR, in patients after myocardial infarction reperfusion (Sarwar et al., 2009). Differences between MDCT and CMR-derived EF was 1%. LV global function among patients referred for coronary angiography was evaluated by Greupner et al., who compared CMR, MDCT, and 2D Echo (biplane Simpson’s rule) and 3D ECHO (Greupner et al., 2012). They concluded that Both MDCT (Multi-Detector Computed Tomography) and 2D Echocardiography had a high level of agreement with CMR, whereas the 3D Echocardiography method showed higher variability when compared to CMR for assessing EF. Furthermore, the measurements of LV volumes and LVEF depend on the geometry of the LV, which might be challenging to obtain these parameters in patients with various heart pathologies. Squeri et al. conducted an interesting study on 66 patients with different heart diseases (including arrhythmogenic right ventricular dysplasia, hypertrophic cardiomyopathy, dilated cardiomyopathy, myocarditis and acute coronary syndrome with normal coronary arteries) with altered chamber geometry (Squeri et al., 2017). They tried to compare real-time three-dimensional echocardiography and 64-slice CT measurements of LV size and function to cardiac MRI in a real-world population. According to their results, RT3DE showed a good linear relationship with the MRI, which reflected in the high correlation coefficients for EDV, ESV and EF, whereas CT displayed less linear relationship with MRI in terms of volume and EF measurements, reflected in the lower correlation coefficients. At the same time, interestingly no statistical difference was found between RT3DE and MRI in different cardiomyopathies. In more recent studies (Tak et al., 2020; Nazir et al., 2024), measurements of EF by CMR and Echo were investigated in detecting and monitoring cardiotoxicity in cancer patients (Tak et al., 2020; Nazir et al., 2024). In both studies, authors concluded that CMR appeared superior to ECHO in detecting early LV systolic dysfunction, thus early cardiotoxicity.

While discussing LVEF as a diagnostic parameter, repeatability of the measurements should be considered as it determines accuracy of the modality. Repeatability refers to the variation of the measurements, when repeated under the same conditions, and should be assessed to ensure that the obtained measurements are coherent for making clinical decisions. Repeatability of the measurements might differ among modalities as a result of methodology (e.g., papillary muscles included in the volume of the left ventricle or in the volume of the myocardium), imaging protocols (e.g., manual, automated or semi-automated) or other key factors including operator skills and experience (e.g., intra- and inter-observer variability), patient factors (e.g., the presence of heart diseases such as myocarditis, cardiomyopathy, heart failure or arrhythmia), contrast medium administration, image quality (Foley et al., 2012). Each modality has its limitations, which directly affects reliability of the measurements. In ECHO factors decreasing reliability of the ejection fraction measurements might be physiological and technical (Bunting et al., 2019; Baysan and Akyıldız, 2019). Physiological factors include load dependency affected by very high or low heart rate, irregular rhythms (e.g., atrial fibrillation) and conduction problems (e.g., left bundle branch block). Technical factors involve poor image quality due to obesity or chronic obstructive lung disease and incorrect geometric assumptions due to a distorted ventricular shape in ischemic heart disease. Then, reproducibility of measurements might be increased by contrast administration in 2D ECHO or by implementing 3D mode, which in addition has been proved to be highly correlated with CMR measurements of LVEF (Wood et al., 2014). In a more recent study by Sveric et al., the authors compared reliability, repeatability, and time efficiency of LVEF measurements between ECHO analyzed by cardiologists with the modified biplane Simpson (MBS) method and by the AI (Sveric et al., 2023). Interestingly, the AI provided a more consistent measurement of LVEF, with a coefficient of variability of 3.2% compared to the MBS method (COV = 5.9%), which opens a discussion on the use of the AI to increase reproducibility of LVEF measurements in the future, diminishing the influence intra- and interobserver variability. CCT and CMR offer quite repetitive LVEF measurements due to high contrast and spatial resolution images, which results in a well-defined endocardial border (Rosenberg and Patil, 2019; Kurtz et al., 2006). Both modalities present few similar limitations that may decrease repeatability of the results. Namely, they both technically depend on ECG gating for image reconstruction and breath holding during image acquisition. As a result, image quality will be reduced in patients with cardiac arrhythmias or ectopic beats and patients with breathing problems or difficulty in following instructions during the exam (such as elderly patients with hearing loss or dementia). In addition to that, in CCT the contrast bolus timing must be on point to ensure proper enhancement of the left ventricle. According to our research, the topic of the LVEF measurements repeatability should be further investigated, since contemporary literature puts more focus on variability and reproducibility of the LVEF measurements, seemingly similar but still different terms. Furthermore, we do agree with authors, who advocate the standardization of the measurement of left ventricular ejection fraction (Kusunose et al., 2001).

The study demonstrates both notable strengths and certain limitations that should be considered when interpreting the findings. The use of three major cardiac imaging modalities—echocardiography, cardiac computed tomography (CCT), and cardiac magnetic resonance (CMR)—allows for a comprehensive comparison of left ventricular ejection fraction (LVEF) assessment, with CMR appropriately serving as the gold standard. The methodology was clearly defined and aligned with current guidelines, and the inclusion of two distinct CCT-based techniques (CCT1 and CCT2) adds depth to the analysis. Additionally, the use of coefficient of variation (CV) to evaluate inter-modality variability provides a quantitative measure of consistency, while the correlation of LVEF variability with patient-specific factors such as body mass index and heart rate adds further clinical insight. Additionally, the results of the study were analysed by the professionals (radiologists and cardiologists) experienced and certified in the evaluation of the cardiovascular system. The study also has some limitations. It lacks correlation of LVEF differences with clinical outcomes, such as functional status or prognosis, which would enhance its practical implications. Furthermore, while the sample size is acceptable for general comparisons, it may be underpowered for subgroup analyses. The absence of interobserver variability assessment also limits understanding of measurement reproducibility. Lastly, the use of CCT raises concerns regarding radiation exposure, which the study does not address. Overall, the strengths of the study support its conclusions, though its limitations should be considered when applying the findings to broader clinical practice.

## 5 Conclusion

1. There are statistically significant differences in left ventricular ejection fraction measurements in patients with a history of SARS-CoV-2 infection using different cardiac imaging modalities. Cardiac computed tomography overestimates LVEF compared to echocardiography and cardiac magnetic resonance imaging.
2. Patients with abnormal body mass, suboptimal heart rate and reduced left ventricular systolic function are subgroups with increased variability of LVEF measurements in different cardiac imaging modalities.

## Data Availability

The raw data supporting the conclusions of this article will be made available by the authors, without undue reservation.

## References

[B1] Abbasi J. (2021). Researchers investigate what COVID-19 does to the heart. JAMA 325 (9), 808–811. 10.1001/jama.2021.0107 33566089

[B2] Artico J. Shiwani H. Moon J. C. Gorecka M. McCann G. P. Roditi G. (2023). Myocardial involvement after hospitalization for COVID-19 complicated by troponin elevation: a prospective, multicenter, observational study. Circulation 147 (5), 364–374. 10.1161/CIRCULATIONAHA.122.060632 36705028 PMC9889203

[B3] Baysan O. Akyıldız İ. (2019). Looking beyond ejection fraction: what we have in echocardiography. Heart, Vessels Transplant. 3, 143. 10.24969/hvt.2019.165

[B4] Bhatt A. S. Kosiborod M. N. Claggett B. L. Miao Z. M. Vaduganathan M. Lam C. S. P. (2023). Impact of COVID-19 in patients with heart failure with mildly reduced or preserved ejection fraction enrolled in the DELIVER trial. Eur. J. Heart Fail 25 (12), 2177–2188. 10.1002/ejhf.3043 37771274

[B5] Bozkurt B. Coats A. J. Tsutsui H. Abdelhamid M. Adamopoulos S. Albert N. (2021). Universal definition and classification of heart failure: a report of the heart failure society of America, heart failure association of the European society of cardiology, Japanese heart failure society and writing committee of the universal definition of heart failure. J. Card. Fail 27, 387–413. 10.1016/j.cardfail.2021.01.022 33663906

[B6] Bunting K. V. Steeds R. P. Slater K. Rogers J. K. Gkoutos G. V. Kotecha D. (2019). A practical guide to assess the reproducibility of echocardiographic measurements. J. Am. Soc. Echocardiogr. 32 (12), 1505–1515. 10.1016/j.echo.2019.08.015 31653530

[B7] Chung M. K. Zidar D. A. Bristow M. R. Cameron S. J. Chan T. Harding C. V. (2021). COVID-19 and cardiovascular disease: from bench to bedside. Circ. Res. 128 (8), 1214–1236. 10.1161/CIRCRESAHA.121.317997 33856918 PMC8048382

[B8] Crosier R. Kafil T. S. Paterson D. I. (2023). Imaging for cardiovascular complications of COVID-19: cardiac manifestations in context. Can. J. Cardiol. 39 (6), 779–792. 10.1016/j.cjca.2023.01.022 36731604 PMC9886397

[B9] Diaz-Navarro R. A. Kerkhof P. L. M. (2024). Cardiac ejection fraction as a problematic metric for heart failure phenotyping. Br. J. Cardiol. 31 (2), 019. 10.5837/bjc.2024.019 39575440 PMC11580666

[B10] Dorosz J. L. Lezotte D. C. Weitzenkamp D. A. Allen L. A. Salcedo E. E. (2012). Performance of 3-dimensional echocardiography in measuring left ventricular volumes and ejection fraction: a systematic review and meta-analysis. J. Am. Coll. Cardiol. 59 (20), 1799–1808. 10.1016/j.jacc.2012.01.037 22575319 PMC3773600

[B11] Foley T. Mankad S. Anavekar N. Bonnichsen C. Miller M. Morris T. (2012). Measuring left ventricular ejection fraction – techniques and potential pitfalls. Eur. Cardiol. Rev. 8, 108. 10.15420/ecr.2012.8.2.108

[B12] Greupner J. Zimmermann E. Hamm B. Dewey M. (2012). Automatic vs semi-automatic global cardiac function assessment using 64-row CT. Br. J. Radiol. 85 (1015), e243–e253. 10.1259/bjr/65747000 22045953 PMC3474063

[B13] Heart Organization (2025). Ejection fraction heart failure measurementa. Available online at: https://www.heart.org/en/health-topics/heart-failure/diagnosing-heart-failure/ejection-fraction-heart-failure-measurement.

[B14] Hoffmann R. von Bardeleben S. ten Cate F. Borges A. C. Kasprzak J. Firschke C. (2005). Assessment of systolic left ventricular function: a multi-centre comparison of cineventriculography, cardiac magnetic resonance imaging, unenhanced and contrast-enhanced echocardiography. Eur. Heart J. 26 (6), 607–616. 10.1093/eurheartj/ehi083 15618026

[B15] Holby S. N. Richardson T. L. Jr Laws J. L. McLaren T. A. Soslow J. H. Baker M. T. (2023). Multimodality cardiac imaging in COVID. Circ. Res. 132 (10), 1387–1404. 10.1161/CIRCRESAHA.122.321882 37167354 PMC10171309

[B16] Kerkhof P. L. M. Kuznetsova T. Ali R. Handly N. (2018). Left ventricular volume analysis as a basic tool to describe cardiac function. Adv. Physiol. Educ. 42 (1), 130–139. 10.1152/advan.00140.2017 29446315

[B17] Kinno M. Nagpal P. Horgan S. Waller A. H. (2017). Comparison of echocardiography, cardiac magnetic resonance, and computed tomographic imaging for the evaluation of left ventricular myocardial function: part 1 (global assessment). Curr. Cardiol. Rep. 19 (1), 9. 10.1007/s11886-017-0815-4 28176279

[B18] Kosaraju A. Goyal A. Grigorova Y. Makaryus A. N. (2025). Left ventricular ejection fraction. Treasure Island (FL): StatPearls Publishing. 29083812

[B19] Kurtz C. E. Gerber Y. Weston S. A. Redfield M. M. Jacobsen S. J. Roger V. L. (2006). Use of ejection fraction tests and coronary angiography in patients with heart failure. Mayo Clin. Proc. 81 (7), 906–913. 10.4065/81.7.906 16835970

[B20] Kusunose K. Zheng R. Yamada H. Sata M. (2001)2022). How to standardize the measurement of left ventricular ejection fraction. J. Med. Ultrason. 49 (1), 35–43. 10.1007/s10396-021-01116-z 34322777 PMC8318061

[B21] Marwick T. H. (2018). Ejection fraction pros and cons: JACC state-of-the-art review. J. Am. Coll. Cardiol. 72 (19), 2360–2379. 10.1016/j.jacc.2018.08.2162 30384893

[B22] McDonagh T. A. Metra M. Adamo M. Gardner R. S. Baumbach A. Böhm M. (2021). 2021 ESC guidelines for the diagnosis and treatment of acute and chronic heart failure. Eur. Heart J. 42 (36), 3599–3726. 10.1093/eurheartj/ehab368 34447992

[B23] Nazir M. S. Okafor J. Murphy T. Andres M. S. Ramalingham S. Rosen S. D. (2024). Echocardiography versus cardiac MRI for measurement of left ventricular ejection fraction in individuals with cancer and suspected cardiotoxicity. Radiol. Cardiothorac. Imaging 6 (1), e230048. 10.1148/ryct.230048 38206164 PMC10912891

[B24] Roger V. L. (2021). Epidemiology of heart failure: a contemporary perspective. Circ. Res. 128 (10), 1421–1434. 10.1161/CIRCRESAHA.121.318172 33983838

[B25] Rosenberg R. D. Patil P. V. (2019). Multimodality imaging of the left ventricle: choosing soundly. J. Nucl. Cardiol. 26, 1865–1868. 10.1007/s12350-018-1294-8 29752640

[B26] Salerno M. Sharif B. Arheden H. Kumar A. Axel L. Li D. (2017). Recent advances in cardiovascular magnetic resonance: techniques and applications. Circ. Cardiovasc Imaging 10 (6), e003951. 10.1161/CIRCIMAGING.116.003951 28611116 PMC5777859

[B27] Sarwar A. Shapiro M. D. Nasir K. Nieman K. Nomura C. H. Brady T. J. (2009). Evaluating global and regional left ventricular function in patients with reperfused acute myocardial infarction by 64-slice multidetector CT: a comparison to magnetic resonance imaging. J. Cardiovasc Comput. Tomogr. 3 (3), 170–177. 10.1016/j.jcct.2009.05.002 19527893

[B28] Savarese G. Becher P. M. Lund L. H. Seferovic P. Rosano G. M. C. Coats A. J. S. (2023). Global burden of heart failure: a comprehensive and updated review of epidemiology. Cardiovasc Res. 118 (17), 3272–3287. 10.1093/cvr/cvac013 35150240

[B29] Squeri A. Censi S. Reverberi C. Gaibazzi N. Baldelli M. Binno S. M. (2017). Three-dimensional echocardiography in various types of heart disease: a comparison study of magnetic resonance imaging and 64-slice computed tomography in a real-world population. J. Echocardiogr. 15 (1), 18–26. 10.1007/s12574-016-0315-3 27589871

[B30] Sveric K. M. Botan R. Dindane Z. Winkler A. Nowack T. Heitmann C. (2023). Single-site experience with an automated artificial intelligence application for left ventricular ejection fraction measurement in echocardiography. Diagn. (Basel) 13 (7), 1298. 10.3390/diagnostics13071298 37046515 PMC10093353

[B31] Tak T. Jaekel C. M. Gharacholou S. M. Dworak M. W. Marshall S. A. (2020). Measurement of ejection fraction by cardiac magnetic resonance imaging and echocardiography to monitor doxorubicin-induced cardiotoxicity. Int. J. Angiol. 29 (1), 45–51. 10.1055/s-0039-1697921 32132816 PMC7054057

[B32] Tański W. Gać P. Chachaj A. Sobieszczańska M. Poręba R. Szuba A. (2021). Selected clinical parameters and changes in cardiac morphology and function assessed by magnetic resonance imaging in patients with rheumatoid arthritis and ankylosing spondylitis without clinically apparent heart disease. Clin. Rheumatol. 40 (11), 4701–4711. 10.1007/s10067-021-05777-6 34173901 PMC8519900

[B33] WHO (2025). World health organization. Available online at: https://www.who.int/news-room/fact-sheets/detail/cardiovascular-diseases-(cvds.

[B34] Wood P. W. Choy J. B. Nanda N. C. Becher H. (2014). Left ventricular ejection fraction and volumes: it depends on the imaging method. Echocardiography 31 (1), 87–100. 10.1111/echo.12331 24786629 PMC4231568

[B35] World Heart Report (2023). Confronting the world's number one killer. Geneva, Switzerland: World Heart Federation.

